# Factors associated with depression among war-affected population in Northeast, Ethiopia

**DOI:** 10.1186/s12888-024-05812-1

**Published:** 2024-05-21

**Authors:** Tamrat Anbesaw, Mulat Awoke Kassa, Wondossen Yimam, Altaseb Beyene Kassaw, Mekonnen Belete, Amare Abera, Gashaw Abebe, Nega Yimer, Mamaru Melkam, Getinet Ayano

**Affiliations:** 1 Department of Psychiatry, College of Medicine and Health Science, Wollo University, P.O. Box 1145, Dessie, Ethiopia; 2 Department of Nursing, College of Health Sciences, Woldia University, Woldia, Ethiopia; 3 Department of Comprehensive Nursing, College of Medicine and Health Sciences (CMHS), Wollo University (WU), P.O. Box 1145, Dessie, Ethiopia; 4 College of Medicine and Health Science, Department of Biomedical Science, Wollo University, P.O. Box 1145, Dessie, Ethiopia; 5 College of Medicine and Health Science, School of Medicine, Department of Physiology, Wollo University, P.O. Box 1145, Dessie, Ethiopia; 6 College of Medicine and Health Science, School of Medicine, Department of Biochemistry, Wollo University, P.O. Box 1145, Dessie, Ethiopia; 7 Department of Adult Health Nursing, College of Medicine and Health Sciences (CMHS), Wollo University (WU), P.O. Box 1145, Dessie, Ethiopia; 8 Department of Psychiatry, University of Gondar College of Medicine and Health Sciences, PO. Box 196, Gondar, Ethiopia; 9 School of Population Health, Faculty of Health Sciences, Curtin University, Kent Street, Bentley, WA Australia

**Keywords:** Depression, Dessie, War, Northeast, Ethiopia

## Abstract

**Background:**

Depression is the most common mental health outcome of exposure to war-related traumatic stressors. Due to inter-communal conflict, Dessie City residents have experienced prolonged armed conflict in 2021. This conflict leads to widespread violence, negative impact on mental health, and large-scale forced migration. However, the problem is not properly addressed in Ethiopia. Therefore, this study aimed to assess the prevalence and risk factors of depression in the war-affected area in Dessie City, Ethiopia.

**Method:**

A cross-sectional study design was conducted among 785 participants in 2022. The study subjects were selected using a multi-stage cluster sampling technique. The outcome measures used in the study were validated with the Patient Health Questionnaire (PHQ-9). Data was entered using Epi-data version 3.1 and SPSS version 25 was used to analyze data. Bivariate and multivariable logistic regressions were done to identify factors related to depression. In multivariable logistic regression variables with a *p*-value less than 0.05 were considered significant and, adjusted OR (AOR) with 95% CI was used to present the strength of the association.

**Result:**

The prevalence of depression among participants was found to be 24.5% (95% CI,21.7, 27.5). In multivariable analysis, post-traumatic stress disorder (AOR = 2.79, 95% CI 1.76–4.43), middle-perceived life threats (AOR = 8.25, 95% CI 2.47–17.49), low social support (AOR = 1.90, 95% CI 1.23–2.96) were variables significantly associated with depression.

**Conclusion:**

This study found a high prevalence of depression among Dessie City residents. post-traumatic stress disorder, middle-perceived life threats, and low social support were associated with depression. Interventional strategies should be implemented to promote healing, resilience, and the overall well-being of individuals and communities. However, the findings underscore the need to address the current lack of mental health care resources in post-conflict populations.

## Background

Depression is defined as a common mental illness that is characterized by persistent sadness, loss of interest in activities that one usually enjoys, feelings of guilt or low self-worth, disturbed sleep or appetite, difficulty performing daily tasks, decreased energy, and poor concentration according to the Diagnostic and Statistical Manual of Mental Disorders, Fifth Edition (DSM-5) [1]. These issues have the potential to become persistent or recurrent, which can seriously affect a person’s capacity to carry out daily responsibilities after being exposed to stressors related with conflict [2]. Suicide is the most common complication of depression, killing an estimated 1 million individuals each year [3].

According to estimates from the World Health Organisation (WHO), mental health problems account for 14% of the world’s disease burden, making them a significant public health concern [4]. Numerous research on the mental health of people impacted by conflict and refugees have been carried out recently; the results show a wide range in the rates of depression (3–85.5%) [5–8] and anxiety (6–72.2%) [9]. In the upcoming ten years, it is anticipated that the financial cost of stress-related mental disease will increase globally [10]. The next ten years are expected to see an increase in the economic burden and global risk of physical illness (obesity, diabetes, pain, etc.) associated to trauma-related mental illness, which could result in disability and lower quality of life.

In war-affected and conflict-ridden peoples, the prevalence rates in the general population can be much higher [11]. According to statistical estimates, the prevalence of depression among people affected by armed conflicts ranges from 2.3 to 80% [6]. The worst civil war was held between the Ethiopian National Defence Forces (ENDF) and The Tigray People’s Liberation Front (TPLF) party that attracted the world’s attention in 2021 [12]. As a result of this conflict; thousands of people lost their lives, and many people were injured and were subjected to traumatic events due to this conflict while they were trying to survive. In addition, violent threats involving rape, torture, mutilation, and destruction of public property facilities such as hospitals, schools, banking and financial sectors, colleges, and many others resulted in the displacement of nearly 2 million people into internally displaced people’s camps (IDPs). Many of these individuals, including children, were abducted, making them more vulnerable to psychological disorders, particularly depression [12]. Furthermore, armed conflicts are linked to poverty, unemployment, communal violence, unstable living conditions, and changes in the social dynamic. This makes the post-war scenario strongly linked to a lower quality of life, which leads to different mental health issues [13].

Research has shown a high prevalence of depression among war-affected populations. According to a meta-analysis study conducted on a worldwide population of war survivors, the prevalence of depression was found to be 26.4% [5]. Also, a survey conducted among Syrian refugees residing in the Kurdistan region of Iraq reported prevalence of depression was 59.4% [6]. In another research conducted on Nepal people who were against the government of Nepal, the estimated prevalence of depression post-conflict situation was 27.5% [7]. Moreover, studies showed in Africa, internally displaced victims in Mogadishu-Somalia 59% [14], Southern Sudan 50% [15], and Uganda was 67% [8]. One cross-sectional study conducted on Somali refugees in Southeast Ethiopia, Melkadida camp reports the overall prevalence rate of depression was 38.3% [16]. Various factors such as being female, lower educational level, alcohol user, chewing khat, having property being destroyed during the conflict, witnessing the murderer of family or friend, being exposed to an increased number of cumulative traumatic events, lack of basic necessities such as food and water, and absence of medical care, personal history of mental illness were significantly associated with depression [17–19]. Additional factors such as anxiety, high perceived life threats, being unemployed after the war, serious physical injury during the conflict, and inadequate social interaction increase the likelihood of developing [20–26].

Over the past decade, conflicts have claimed over three times more lives than natural disasters [27]. Due to these a high prevalence of depression among war-affected populations highlights the need for mental health interventions that address the specific needs of these populations. Also, a need for governments and international organizations to prioritize mental health services for war-affected populations. Additionally, the negative impact of war on mental health highlights the need for policies that address the root causes of war and promote peace and stability [28]. In Ethiopia, the rising incidents of armed conflict and ethnic violence present a unique context, necessitating a closer examination of the mental health challenges faced by the affected population. Despite the increasing evidence of high depression rates in conflict-affected nations worldwide, the scarcity of studies focusing on Ethiopia’s situation leaves a critical gap in understanding the mental health dynamics specific to this region [22]. Therefore this study aimed to assess the prevalence of depression and its associated factors among war affected population in Dessie City situated in the area of armed conflict. The findings of this study will help medical practitioners, NGOs, and psychological centers to develop appropriate plans and interventions to provide evidence-based treatment for patients with depression. Additionally, it will also provide baseline data for future studies and researchers.

## Methods and materials

### Study design and period

A population-based cross-sectional study was conducted in selected war-affected areas from June 8- July 7/2022.

### Study area

Dessie City is one of the administrative centers in the Amhara Region; located 401 Km from Addis Ababa to Northeast Ethiopia. It is located at latitude 11°8′N, longitude 39°38′E, and height 2,470–2,550 m above sea level. It has 350,000 residents and 18 kebeles. Data from the South Wollo Zone statistics office from 2016 to 2017 show that there were 163,429 females and 186,571 males in those populations. Psychiatric inpatient and outpatient services are available at the two government facilities.

### Source of population

All residents of Dessie city administration, North East, Ethiopia.

### Study population

Households residing in the kebele of the Menafesha sub-city who are in the selected kebele and available during the study period.

### Inclusion and exclusion criteria

#### Inclusion criteria

All households in the selected kebeles as well as adult residents who were *≥* 18 years old at the time of the study were included.

#### Exclusion criteria

Those participants who were not present during wartime were unable to communicate and were critically ill during data collection time, those residents less than 6 months were excluded from the study.

### Sample size determination and sampling technique

#### Sample size determination

A 50% proportion was used to calculate the sample size, since there is no related study done to identify the magnitude of depression in Ethiopia war affected area. The sample size was estimated by using a single population proportion formula. Sample size with z-value of 1.96 and marginal error of 5% sample was calculated as:

$${\rm{n}}\,{\rm{ = }}\,\frac{{{{\left( {{\rm{Z}}\alpha {\rm{ /2}}} \right)}^{\rm{2}}}{\rm{x P}}\,\left( {{\rm{1}} - {\rm{P}}} \right)}}{{{{\rm{d}}^{\rm{2}}}}}$$

Where: *n* = sample size, *α* = confidence interval (95%) *p* = proportion of = 50% (0.5).

$${\rm{d = marginal}}\,{\rm{error}}\,{\rm{of}}\,{\rm{5\% ,}}\,{\left( {{\rm{z}}\,{\rm{\alpha }}{{\rm{/}}_{\rm{2}}}} \right)^{\rm{2}}}{\rm{ = 1}}{\rm{.96}}$$

$${\rm{n = }}\,\frac{{{\rm{1}}{\rm{.9}}{{\rm{6}}^{\rm{2}}}{\rm{x}}\,{\rm{(0}}{\rm{.5}}\,\left( {{\rm{1 - 0}}{\rm{.5}}} \right)}}{{{{\left( {{\rm{0}}{\rm{.05}}} \right)}^{\rm{2}}}}}\,{\rm{ = 384}}$$

Since we have employed a multi-stage sampling technique we consider the designing effect by calculated sample size by 2 to correct the sampling error. After all, the final sample size was 768. We added a 5% [38] non-response rate; finally, the total sample size was 806.

#### Sampling technique and procedure

A probability sampling technique with multiple stages was utilized. Dessie City has five sub-cities. One sub-city was chosen at random from the other five using a basic random sampling technique. This sub-city has three kebeles, so the number of participants was determined by allocating the sample size proportionately to each kebele. Then, to choose the study units, a methodical random selection procedure was used. The primary study unit and study subjects were chosen at random from every K^th^ family between the first and the K^th^. When more than one research subject was discovered in a single home, a volunteer was chosen by lottery. The next household member was interviewed in the event that the selected home was closed at the time of data collection. This implies, Kj = Nj/nj, where Nj is the total number of homes, Kj is the sampling interval, and nj is the total sample size. The Menafesha sub-city consists of three kebeles with a total of 3,832 households; K, or the interval, was determined by N/n (K = 3832/821 = 4). Participants included from the subcity Nebsu hager 971(214), Kelem meda 1 265(279), and Tekuam 1596(352). They conducted sequential interviews with one person from each of the four families.

### Data collection method and technique

Data were collected by face-to-face interviews using a structured questionnaire. The Patient Health Questionnaire (PHQ-9) was used to assess the presence of depression, with a score of 10 or higher indicating depression [29]. The validity and reliability of the PHQ-9 have been assessed and confirmed for use in community studies in a number of countries [22]. The internal consistency (Cronbach alpha) of (PHQ-9) was 0.84. The 20-item Post-Traumatic Checklist (PCL-5) was used to measure PTSD with ratings ranging from 0 to 80 on a 5-point Likert scale (0 = Not at all, 1 = A little bit, 2 = moderately, 3 = Quite a bit, and 4 = excessively). A score of ≥ 33 was used to define symptoms of PTSD [30]. Numerous countries have investigated and validated the validity and reliability of the PCL-5, including Zimbabwe (Cronbach’s alpha = 0.92) [31] and Iraq (Cronbach’s alpha = 0.85) [32]. In this study, the PCL-5s’ internal consistency (Cronbach alpha) was 0.88. The perceived life threats were measured using the 0–40 range of the perceived stress (PSS) scale. The PSS indicated that respondents who scored between 0 and 13 felt low perceived stress, those who scored between 14 and 26 felt moderate perceived stress, and those who scored between 27 and 40 felt high perceived stress [33]. It has an internal consistency (Cronbach alpha) value of 0.89. The Oslo 3-item Social Support Scale (OSSS-3) was utilized to gather information about the degree of social support. It was categorized into three broad categories of social support; poor social support [3–8], moderate social support [9–11], and strong social support [12–14, 34]. Substances were measured using the WHO student drug-use questionnaire [35]. Response questionnaires, sociodemographic characteristics, past drug use, clinical considerations, and trauma-related factors on “yes/no” questions. These factors were operationalized in accordance with various literary works.

#### Data collection procedures

To ensure consistency, the questionnaire was written in English at first, translated into Amharic, and then returned to English. In addition, we provide supervisors and data collectors with two days of training before using conventional methods to determine the outcome variable. 5% (*n* = 42) of the participants in the Hotie sub-city completed the pre-test, which was designed to find any possible problems with the data collection techniques and make recommendations for modifications to the survey. On a regular basis, the supervisor and lead investigator provided guidance and support to the data collectors. Supervisors and principal investigators checked the data for consistency and completeness each day during the data collection period.

#### Data processing and analysis

Epi-data version 3.1 was used to check and clean the data before entering them into the computer system. After that, the data were exported to SPSS version 26 statistical software for further analysis. Frequency, percentage, and other descriptive statistics were used by the researchers. Independent variables with a bivariable model *p*-value of less than 0.25 were included in the multivariable regression model to consider potential confounding effects. The model of fitness was checked by Hosmer and Lemeshow Goodness. For every variable in the multivariable model with a *p*-value of less than 0.05, an odds ratio with a 95% confidence interval showed the strength of association. The final result was to report the findings in text, a table, or a graph. Variance inflation factors and tolerance were checked to test multicollinearity or to see the unique effect of predictors on outcome variables.

## Results

### General characteristics of the study population

In this study, a total of 785 participants were assessed, with an overall 93.01% of response rate. The mean (SD) age of the participants was 36.01 (± 11.29) years. More than half (56.9%) of respondents were male. Almost two-thirds of the participants (66.4%) were married. The majority 203(25.9%) of the participants had attended a University degree and above. The majority of the community were 249(31.7%) merchants by occupation. More than half of 460(58.6%) participants reported that their average monthly income is above 2166 Ethiopian birr (Table 1).

**Table 1:** Socio-demographic characteristics of participants

Variables	Category	Frequency	Percentage (%)
Age	18–24	83	10.6
	25–34	297	37.8
	35–44	243	31.0
	> 44	162	20.6
Sex	Male	447	56.9
	Women	338	43.1
Marital status	Lack of cohabiting partner	264	33.6
	Married	521	66.4
Educational status	Unable to read and write	114	14.5
	Primary school	109	13.9
	Secondary school(9–12 grade)	171	21.8
	College diploma	188	23.9
	University degree and above	203	25.9
Occupational status	Government employee	224	28.5
	Housewife	111	14.1
	Merchant	249	31.7
	Student	119	15.2
	Others*	82	10.4
Average monthly income (Eth. Birr)	< 2166	325	41.4
	>=2166	460	58.6

Others*:- NGOs, Retired, & Farmer

### Clinical-related factors of the participants

The current study found that 40 (5.1%) of the respondents had previously experienced mental illness. Of the participants, 39 (5.0%) reported a family history of mental illness and 88 (11.2%) reported a history of chronic medical illness. Also, this finding shows that of the individuals, 262 (33.4%) reported symptoms of anxiety. Regarding substance usage, 186(23.7%) had ever drunk alcohol, 196(25%) had used khat, and 8.7% had smoked cigarettes in their lifetime. While 125(15.9%) drank alcohol, 134(17.1%) use khat and 43(5.5%) of the respondents smoke cigarettes currently (Table 2).

**Table 2:** Description of clinical and related factors of respondents

Variables	Category	Frequency	Percentage (%)
History of mental illness	Yes	40	5.1
	No	745	94.9
Family history of mental illness	Yes	39	5.0
	No	746	95.0
History of chronic medical illness	Yes	88	11.2
	No	697	88.8
Anxiety	Yes	262	33.4
	No	523	66.6
Ever alcohol use	Yes	186	23.7
	No	599	76.3
Current alcohol use	Yes	125	15.9
	No	660	84.1
Lifetime khat use	Yes	196	25.0
	No	589	75.0
Current khat use	Yes	134	17.1
	No	651	82.9
Lifetime cigarette use	Yes	8.7	68
	No	91.3	717
Current cigarette uses	Yes	43	5.5
	No	742	94.5

### Trauma-related and psychosocial factors

In terms of personal trauma, the kind of trauma that the community has experienced the major physical injuries sustained during the conflict witnessed deaths in the family, major bodily injuries sustained by family members or friends, and property damaged during the fighting occurred in 72 (9.2%), 33 (4.2%), 66 (8.4%), and 116 (14.8%) cases, respectively. From this study, 120 (15.3%) had reported suffering childhood maltreatment, 357(45.5%), had moderate perceived life threats and 420(53.5%) had received threat intermittent social support. Of the participants, 152(19.4%) had experienced post-traumatic stress disorder (Table 3).

**Table 3:** Description of trauma-related and psychosocial factors

Variables	Category	Frequency	Percentage (%)
Serious physical injury at the time of conflict	Yes	72	9.2
	No	713	90.8
Witnessing death in the family	Yes	33	4.2
	No	752	95.8
Property be damaged	Yes	116	14.8
	No	669	85.2
Childhood abuse	Yes	120	15.3
	No	665	84.7
Perceived life threat	Low perceived life threat	179	22.8
	Moderate perceived life threat	357	45.5
	High perceived life threat	249	31.7
Levels of social support	Low social support	176	22.4
	Medium social support	420	53.5
	High social support	189	24.1
Post-traumatic stress	Yes	152	19.4
	No	633	80.6

### Prevalence of depression

In this study, the overall prevalence of depression among people who experienced traumatic events in Dessie City was 24.5% [ (95% CI,21.7, 27.5)].

### Factors associated with depression

In the bivariate analysis, monthly income, mental illness history, family mental illness history, childhood abuse history, serious physical injury, witnessing the death of a family member, post-traumatic stress, perceived life threat, and low social support showed a *P*-value of < 0.25 and became a candidate for multivariable analysis. Variables such as post-traumatic stress disorder, moderate perceived life threat, and poor social support were found to be significantly associated with depression at a *p*-value less than 0.05 in multivariable analysis.

Those participants who experienced post-traumatic stress disorder were 2.79 times more likely to develop depression than those who had not experienced post-traumatic stress (AOR = 2.79, 95% CI 1.76–4.43). The odds of developing depression among respondents who had experienced middle-perceived life threats were eight times higher than those who had low-perceived life threats (AOR = 8.25, 95% CI 2.47–17.49). Likewise, those participants who received low social support were 1.90 times more likely to develop depression than those who received strong social support (AOR = 1.90, 95% CI 1.23–2.96) (Table 4).

**Table 4:** Bivariate and multivariable logistic regression analysis results of depression participants

Variables	Category	Depression		COR(95%C.I)	AOR(95%C.I)	*P*-values
		Yes(n)	No(n)			
Monthly Income	< 2166	87(25.7%)	251(74.3%)	1.31(0.81,1.56)	0.98(0.68,1.43)	0.938
	>=2166	105(23.5%)	342(76.5%)	1	1	
Mental illness history	Yes	15(37.5%)	25(62.5%)	1.92(0.99,3.733)	1.14(0.45,2.89)	0.78
	No	177 (23.8%)	568(76.2%)	1	1	
Mental illness history family	Yes	14(35.9%)	25(64.1%)	1.787(0.91,3.51)	0.53(0.05,5.22)	0.591
	No	178(23.9%)	568(76.1%)	1	1	
Childhood abuse history	Yes	34(28.3%)	86(71.7%)	1.27(0.82,1.96)	0.95(0.44,2.07)	0.91
	No	158(23.8%)	507(76.2%)	1	1	
Serious physical injury	Yes	25(34.7%)	47(65.3%)	1.74(1.04,2.91)	1.26(0.61,2.60)	0.53
	No	167(23.4%)	546(76.6%)	1	1	
Witness the death of a family member	Yes	14(42.4%)	19(57.6%)	2.37(1.17,4.84)	1.15(0.25,5.39)	0.858
	No	178(23.7%)	574(76.3%)	1	1	
Post-traumatic experiences	Yes	55(36.2%)	97(63.8%)	2.05(1.41,3.01)	2.79(1.76,4.43)	< 0.001*
	No	137(21.6%)	496(78.4%)	1	1	
Perceived life threat	Low perceived life threat	3(1.7%)	176(98.3%)	1	1	
	Moderate perceived life	148(41.5%)	209(58.5%)	11.56(3.52,27.98)	8.25(2.47,17.49)	0.001*
	High Perceived life threat	41(16.5%)	208(83.5%)	0.28(0.18,0.41)	1.12(0.12,1.53)	0.081
Social support	Low social support	57(32.4%)	119(67.6%)	0.62(0.38,0.978)	1.90(1.23,2.96)	0.004*
	Intermittent social support	92(21.9%)	328(78.1%)	1.05(0.69,1.58)	1.64(0.98,2.755)	0.059
	Strong social support	43(22.8%)	146(77.2%)	1	1	

*Statistically significant at *P*-value < 0.05, COR, Crude Odds Ratio, AOR, Adjusted Odds Ratio, 1 = reference category, Hosmer Lemeshow goodness-of-fit 0.52

## Discussion

Even though the prevalence of mental illness growing fast, depression in war-affected areas is not well addressed. Therefore, this study was intended to address this gap by assessing the prevalence and associated factors of depression in the war-affected area in Northeast, Ethiopia. The result of this study showed that the prevalence of depression among people who experienced traumatic events in Dessie City was 24.5% [(95% CI,21.7, 27.5)]. The finding was in line with the study conducted among depressive participants in post-conflict situations in Nepal 27.5% [7], and the meta-analysis study conducted by *Bedaso, et al.* 26.4% [5]. However, the current study was lower when compared with studies done in Iraq 59.4% [6], Southeast Ethiopia 38.3% [16], Mogadishu-Somalia 59% [14], Southern Sudan 50% [15], Syrian refugees 37.4% [36], Uganda 67% [8]. The disparity might be due to the cut score of the assessment tool; for instance, in Southerneast Ethiopia, depression was assessed using a PHQ-9 score with a cut score of ≥ 5 [16], while the current study cut point was a score of PHQ-9 ≥ 10, which was validated instrument in Ethiopia. Another discrepancy includes endorsed to the length of time they have been exposed to these heinous acts and ongoing feelings of insecurity among participants. In our study, participants were subjected to these worse circumstances for a shorter time than Uganda [8], and Southern Sudan [36]. Moreover, all inhabitants are included in our study not simply those living in camps for refugees.

This result was higher than those found in studies in Sri Lanka 5.1% [37], Vietnamese refugees in the United States 20% [38], and Toronto 9.8% [39]. The discrepancy in the instruments could be the cause of this variation; in which structured interviews using DSM-IV-TR in Sri Lanka [40], whereas in this study the PHQ-9 was used, and extended standards criteria. The prevalence of depression could be lower due to the result of more psycho-social support in Toronto [39], and Vietnamese [38]. Another reason might be the duration of exposure to traumatic events; the study was conducted in Sri Lanka after twenty years of forced displacement, but the current study was conducted after six months of exposure to trauma. As a result, extended exposure to the traumatic event was more likely to result in a reduction in magnitude due to recall bias. Furthermore, the disparity may also be due to the various forms of trauma exposure, the sample procedures, social and sociocultural elements, and demographic variables in the populations.

Regarding the associated factors, post-traumatic stress disorder was 2.79 times more likely to develop depression than those who had not experienced post-traumatic stress. This finding was in agreement with different studies in Uganda [8], and Nepal [7]. Individuals who have experienced trauma during the war may develop post-traumatic stress disorder, a condition closely linked to depression. Flashbacks, nightmares, and intrusive memories associated with post-traumatic stress disorder can contribute to depressive symptoms [21].

The odds of developing depression among respondents who had experienced middle-perceived life threats were 8 times higher than those who had low-perceived life. Similar to a finding of different studies from Nepal [7]. Depression symptoms were more severe in individuals who believed that the conflict had a negative effect on their communities than in those who believed it had a good one. This could be due to real-life occurrences, political beliefs, or a demoralizing position that could have an adverse effect on mental health. There is evidence to suggest that having a negative perspective on the conflict is linked to having worse mental health [41].

Likewise, those participants who received low social support were 1.90 times more likely to develop depression than those who received strong social support. Similar to a finding of different studies from Nepal and Southeast Ethiopia [16]. War and conflict can strain social relationships, leading to a lack of support from family, friends, or community. The absence of a strong support system can contribute to feelings of loneliness and exacerbate depressive symptoms.

### Limitations of the study

The use of a high response rate and the addition of important variables were not included in previous studies. Also, PHQ-9 was used for measuring depression, we employed an updated standardized tool and it is validated in Ethiopia. As well, validated and standardized measures were used to assess independent factors such as social support, perceived stress, and post-traumatic stress disorder. On the other hand, because of the cross-sectional study design, it does not allow to infer causation. It is recommended that future researchers do their studies among this affected population as the research did not include the afflicted demographic as children in traumatic events. Moreover, recall bias and social desirability could also be additional limitations.

## Conclusion

This study showed a high prevalence of depression among the war-affected population in Dessie City. Post-traumatic stress disorder, moderate perceived life threat, and poor social support were found to be significant predictors of depression. Therefore, addressing the mental health consequences of war requires a comprehensive and multidimensional approach, including international cooperation, advocacy for mental health awareness, and targeted interventions at individual, community, and systemic levels. It’s crucial to recognize the unique challenges faced by those affected by war and to implement strategies that promote healing, resilience, and the overall well-being of individuals and communities.

### Implications

Addressing depression after war requires a comprehensive approach that includes mental health interventions, social support, and community-based programs. Providing access to mental health services, promoting awareness, and reducing stigma are crucial components of supporting individuals dealing with post-war depression. Additionally, a holistic approach that considers the interconnectedness of mental, emotional, and physical well-being is essential for promoting recovery and resilience.

## Data Availability

All data analyzed during this study are included in this published article.

## Abbreviations

AOR: Adjusted Odds Ratio
CI: Confidence Interval
COR: Crude Odds Ratio
DSM: Diagnostic Statistical Manual
NGOs: Non-governmental organizations
OSSS-3: Social Support Scale
PCL: Post-traumatic Stress Disorder Checklist
PHQ-9: Patient Health Questionnaire
PSS: Perceived Stress Scale
PTSD: Post-traumatic Stress Disorder
SD: Standard deviation
SPSS: Statistical Package for Social Science
WHO: World Health Organization
